# The New Modular Sforzesco Brace (Modular Italian Brace) Is as Effective as the Classical One: A Retrospective Controlled Study from a Prospective Cohort

**DOI:** 10.3390/jcm13072075

**Published:** 2024-04-03

**Authors:** Francesco Negrini, Francesca Febbo, Fabrizio Tessadri, Andrea Zonta, Marta Tavernaro, Sabrina Donzelli, Fabio Zaina, Stefano Negrini

**Affiliations:** 1 Department of Biotechnology and Life Sciences, University of Insubria, 21100 Varese, Italy; francesco.negrini@uninsubria.it; 2 Istituti Clinici Scientifici Maugeri IRCCS, 21049 Tradate, Italy; 3 ISICO (Italian Scientific Spine Institute), 20141 Milan, Italy; andrea.zonta@isico.it (A.Z.); marta.tavernaro@isico.it (M.T.); sab.donzelli@gmail.com (S.D.); fabio.zaina@isico.it (F.Z.); 4 Orthotecnica, 38014 Trento, Italy; fabriziotessadri@gmail.com; 5 Department of Biomedical, Surgical and Dental Sciences, University “La Statale”, 20122 Milan, Italy; stefano.negrini@unimi.it; 6 IRCCS Istituto Ortopedico Galeazzi, 20161 Milan, Italy

**Keywords:** idiopathic scoliosis, brace, conservative treatment, Sforzesco, MI brace

## Abstract

**Background**: The Sforzesco brace is a very rigid push-up brace effective in adolescent idiopathic scoliosis (AIS). We recently developed a new Sforzesco brace based on modularity (the Modular Italian brace—MI brace) that could allow standardization, facilitating global expertise diffusion, increased modifiability and adaptability, and cost savings due to longer brace life. We aimed to compare the short-term results of the two braces. **Methods**: The retrospective study included 231 consecutive AIS treated with a MI brace (N = 53) or Sforzesco brace (N = 178). The main outcome was the first 6-month follow-up out-of-brace radiograph Cobb angle change. Secondary outcomes included the in-brace Cobb degrees and aesthetics (TRACE), prominence (angle of trunk rotation and mm), kyphosis, and lordosis changes. **Results**: The two groups were similar at baseline, apart from more immature patients in MI brace. Both braces reduced the Cobb angle (−6° out-of-brace; −16° in-brace) without differences between groups. All secondary outcomes improved, apart from a statistically and clinically insignificant 3° kyphosis reduction. The MI brace participants were 4.9 times more likely to improve the Cobb angle than the Sforzesco brace (OR = 4.92; 95%CI 1.91–12.64; *p* = 0.001). **Conclusions**: These findings suggest that the MI-brace can be safely used instead of the classical Sforzesco brace. However, further studies of different designs and longer follow-ups are needed to confirm these findings.

## 1. Introduction

Idiopathic scoliosis is a structural three-dimensional deformity of the spine and trunk [1,2,3], and it is the most common spinal deformity during growth. Braces are recommended for moderate to severe idiopathic scoliosis during growth [1]. Good quality studies showed the efficacy of bracing to treat adolescents with idiopathic scoliosis (AIS) [4,5,6,7,8,9], and a Cochrane review summarized the current evidence [10]. Which brace has the best biomechanical action on the scoliotic spine is still unknown, but experts often link biomechanics to different brace designs [11].

Brace efficacy is strongly related to dosage [12,13], but brace-wearing hours are also considered inversely correlated to demand on patients [1,4,14]. Materials, production methods, and personalization of the brace have all evolved in the last decades to improve efficacy and patient comfort [7,15,16,17,18,19,20,21]. It can be postulated that increased comfort could also improve compliance, which is one of the main factors of brace efficacy [15].

The Sforzesco (SfB) is a very rigid brace built according to the SPoRT concept (an acronym for symmetrical, patient-oriented, rigid, three-dimensional, active). It was introduced in the first decade of this millennium, and its effectiveness has been proven, especially in severe curves [10]. Recently, the “Free Pelvis” (FP) innovation has been introduced into the SfB [15]. The FP consists of semi-rigid material (ethylene vinyl acetate) on the pelvis connected to the main very rigid body of the brace (high-density polyethylene) on the trunk. It was found that this innovation, introduced to improve patient comfort and brace adaptability, does not change the in-brace and short-term results of classical very rigid braces and, consequently, can be safely applied [15].

**Figure 1:**
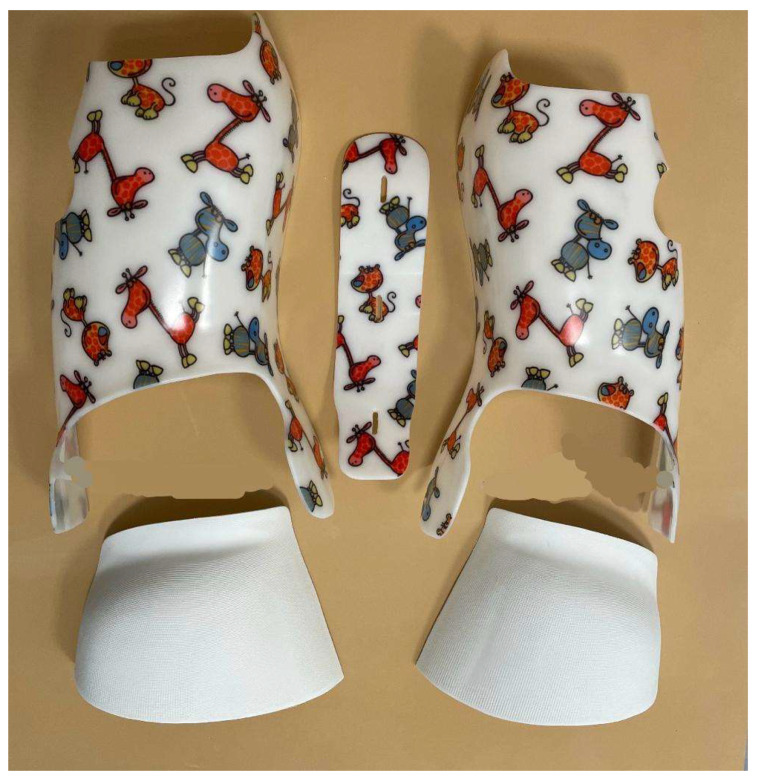
The 5 modules of the Modular Italian brace (MI brace).

The FP innovation allowed the Modular Italian brace (MI brace—MIB) to be developed. This brace combines the FP innovation with an adjustable posterior polyethylene bar (APB) that gives complete modularity to the brace (five modules: two valves, two FPs, and one APB) (Figure 1).

The MIB can be built and fully personalized directly on the patient, modifying the classical procedures of the brace construction through CAD-CAM or casting: body reconstruction, creation of a corrected positive, and moulding. Moreover, the MIB can be continuously adapted during treatment, giving full adaptability to the patient during changes due to growth or treatment.

On that regard, the MIB is an evolution of the Sf FP. The two braces share the semi-rigid material on the pelvis, a novelty that improves comfort and patient acceptability. On the other hand, Sf FP is built in a more traditional way [22], without the MIB modularity and the possibility to adapt the brace during treatment; with the Sf FP, if the patient changes due to growth, the brace must be changed altogether.

From a biomechanical point of view, the three braces share the same corrective principles, that is, the push-up action [11]. The FP does not change the biomechanical action on the spine, but simply offers freedom and comfort to the pelvis. It is possible that it could give a change in the sagittal plane, but this has not yet been explored. The MI brace, even if built in a different way, keeps the main characteristic of the Sf brace, that is, the overall symmetricity (with some asymmetries inserted in specific areas). In fact, it is the symmetricity that allowed the development of the MI brace, where it is possible to re-create the asymmetrical needs of the Sf brace through internal pads.

The current understanding on bracing [11] is that under the same name, it is possible to provide braces with different overall actions, and the (biomechanical) action does not depend on the name of the brace, but on the conception and consequent construction of it. Consequently, the braces considered in this paper share the same action according to the treating team—the question is if they also share the same results.

Before considering any other possible evolution, there is the need to check the MIB effectiveness compared to the original SfB, the current gold standard. This study aims to compare the results of the MIB with those of the SfB in the short term in the largest possible population from the same treating team: physicians, orthotist, and physiotherapist. We considered the first most significant outcome, that is, the first out-of-brace X-rays after 4–6 months of treatment; recent studies have shown that this X-ray allows us to precisely predict the final treatment results (mean difference 1.8 ± 5.2°) [23]. To achieve this aim, we performed a retrospective analysis of all data prospectively collected in a tertiary referral outpatient institute. We looked at the desired outcomes within a cohort of consecutive AIS patients with an SfB prescription, comparing those receiving the original SfB to those receiving the new MIB.

## 2. Materials and Methods

### 2.1. Design

This is a retrospective study of a prospective cohort of consecutive AIS patients. The data were collected in an outpatient referral institute specialized in conservative scoliosis treatment since the development of the SfB. The development of the MIB (Figure 1) gradually caused the discontinuation of the SfB. For this study, we considered only patients treated by the same physicians (AZ, SN) with the brace built by the same orthotist (FT) working in a team with the same physiotherapist (MT). The Local Ethics Committee approved the study (Ethics Committee Comitato Etico Milano Area 2, Via F. Sforza 28, 20122 Milan Italy—parere 466_2021, date of approval: 27 April 2021). Informed consent to the acquisition of anonymized clinical data for analysis was collected from the parents of all the patients involved. The study did not receive any external funding. The study was reported following the STROBE checklist indications [24].

### 2.2. Participants

**Figure 2:**
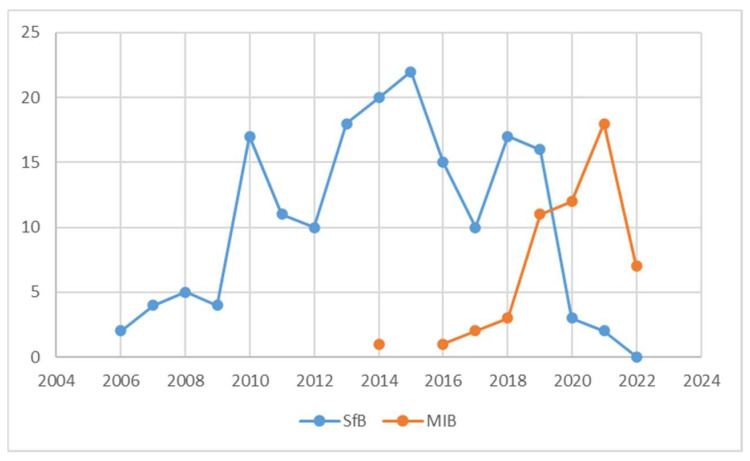
Number and type of prescribed braces per year. SfB: Sforzesco Brace; MIB: Modular Italian brace (MI brace).

We included consecutive adolescents according to the following inclusion criteria: diagnosis of AIS, aged 10 to 16, very rigid SfB prescription at the first consultation, frontal radiographs available at the three time points considered (baseline, in-brace, and out-of-brace at the first 5 ± 2-month medical follow-up). Exclusion criteria were any other previous or current pathology of the spine or neuromuscular and musculoskeletal systems and any disease possibly associated with scoliosis. We collected all the available data corresponding to the inclusion criteria up to 31 December 2022. We divided participants into the SfB and MIB groups, according to the brace prescribed and built, respectively. Figure 2 shows our cohort’s gradual transition from the SfB to the MIB. There are a few years of overlap during the first experimental cases.

### 2.3. Treatments

The SfB was built for every patient with a prescription for a very rigid brace until 2016, when the development of the MIB started with a gradual shift toward the new brace, finally completed in 2022 (Figure 2). The physician and orthotist did not make specific choices in favour of one or the other brace during the overlap period, where random factors like time availability, human resources, and external situations determined the construction of one or the other brace. Most patients performed scoliosis-specific exercises (PSSE) according to the SEAS (the acronym in everyday use for Scientific Exercises Approach to Scoliosis) School [25,26].

**Figure 3:**
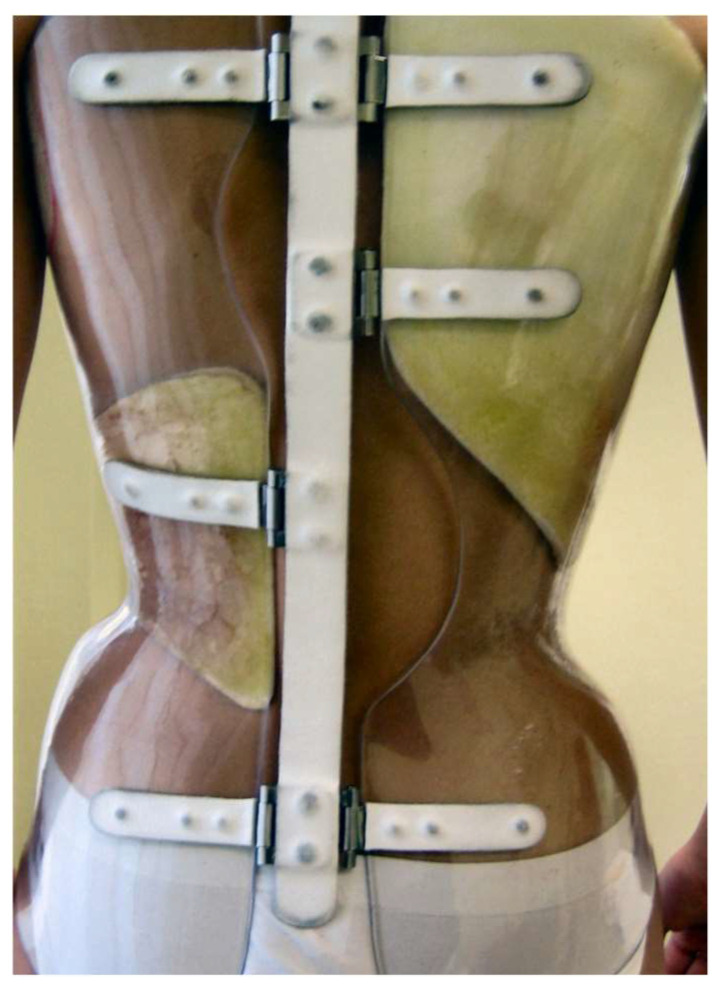
The Sforzesco brace.

The SfB (Figure 3) has already been thoroughly described in previous publications [27]. According to the recently published classification of braces [11], it is a very rigid, push-up, three-dimensional, bivalve, ventral closure, thoraco–lumbo–sacral orthosis.

**Figure 4:**
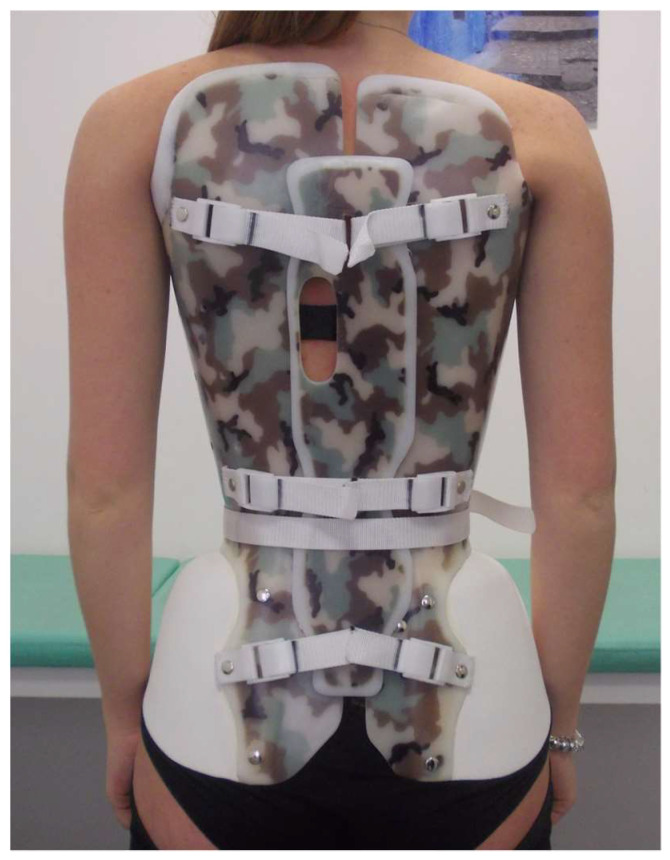
The Modular Italian brace (MI brace).

The MIB is modular (Figure 4), with five modules: the two valves on the trunk are the same as in the SfB, with a difference at the pelvis where the two FP modules substitute the classical rigid material; the 5th module is the APB (Corsetto ortopedico regolabile. Inventors Stefano Negrini and Fabrizio Tessadri. Owners ISICO srl and Fabrizio Tessadri. Patent Ita-102020000003991).

The orthotist chooses the dimensions of the five modules according to a specific series of measurements (lengths, diameters, and circumferences) acquired either through a laser body scanning or rulers and callipers. The orthotist combines the modules directly on the patient when they perform all final refinements according to the correction needs. At this stage, they usually also add internal Plastazote foam pushes. The anterior and posterior tightening and the dimensions of each single module are totally adjustable to provide a complete customization and individualization of the brace. Also, the final refinement of the FP on the pelvis are performed directly on the patient, heating the material on the donned brace. All adjustments are performed at different moments: while providing the final brace to the patient and at the medical brace checks and consultations. This makes the MIB much more flexible and continuously adjustable, compared to the SfB. Technically, also the MIB is a very rigid, push-up, three-dimensional, bivalve, ventral closure, thoraco–lumbo–sacral orthosis [11]. Hence, it remains a SfB, with more comfort at the pelvis (due to the FP), more adjustability (due to the APB), and possibly increased posterior rigidity (due to the superposition of APB on the two trunk modules). It could have reduced stability because the APB is not fixed like the hinges of the SfB and due to the FP. The latter has already been tested and does not negatively impact the short-term clinical results [15].

### 2.4. Outcomes

The main outcomes were the changes in the Cobb angle of the major curves measured on coronal radiographs. We had three observation times: at baseline (T0), while wearing the brace (T1), and after the brace was removed (T2). The T1 radiograph was prescribed to be performed one month after the treatment was started. The T2 radiograph was performed at the first consultation prescribed at six months after the brace prescription; the patient was asked to remove the brace immediately before the X-ray. The Cobb angle of lumbar lordosis and thoracic kyphosis were also evaluated. All radiological assessments were performed following the recommendation of SOSORT guidelines [1]. The reliability of the radiographic measurements was previously verified by clinicians at our institution (5° of inter-observer measurement error) [1,6,28]. The secondary outcomes were clinical assessments of the angle of trunk rotation (ATR) measured using a Bunnell Scoliometer and aesthetics using the Trunk Aesthetic Clinical Evaluation (TRACE) score [29]. Compliance was measured using a heat sensor (Thermobrace) [30]. The treating physicians collected all measurements at baseline and the first visit after six months from the brace prescription.

### 2.5. Potential Confounders

Compliance was measured using a heat sensor (Thermobrace) [30]. Exercise adherence was not measured. Risser at the start of the treatment was included in an appropriate sensitivity analysis.

### 2.6. Statistical Analysis

After checking the normal distribution of the data, we described participants for all clinical and radiographic parameters. Depending on the variable type, we used the unpaired *t*-tests and chi-square to check the differences between the two groups at baseline. We performed an unpaired *t*-test to evaluate the result differences between the two brace designs. We planned to check the odds ratio for improvement with logistic regression for all the differences found. Therefore, we tested the brace design as a predictor of results (change to >5° Cobb angle) with a logistic regression model with adjustment for clinical and radiographic parameters. After checking the univariate model, we tested the following variables in a multivariate model: Cobb angle at baseline, sagittal angles at baseline, clinical measures (TRACE, ATR, and prominence in mm) at baseline, and age at baseline. The alpha level of significance was set at 0.05. For the analysis, we used STATA 15 software (Copyright 1985–2017 StataCorp LLC, College Station, TX 77845, USA). Finally, we performed a sensitivity analysis using logistic regression including only younger patients starting brace treatment with a Risser of 0 to 2.

## 3. Results

This study included 231 patients, 178 in the SfB group and 53 in the MIB group. All clinical, radiological, and demographic data and comparisons between groups are presented in Table 1.

Most patients included in the study were female (85.4% in SfB and 75.5% in MIB), with a mean age at the start of treatment of 13.6 ± 1.7 years in SfB and 13.5 ± 1.9 years in MIB. The mean Cobb angle at the start of treatment was 40.9 ± 12.8° in SfB and 38.6 ± 11.2° in MIB. At baseline, most patients were skeletally immature (Risser 0–2) in both groups (percentage of patients with Risser 0–2 was 63.3% for SfB and 79.2% for MIB). The only difference between the two groups at T0 was in the proportion of skeletal immature (Risser 0–2) patients, which was significantly higher in the MIB group (chi-square: 14, 18). 

**Figure 5:**
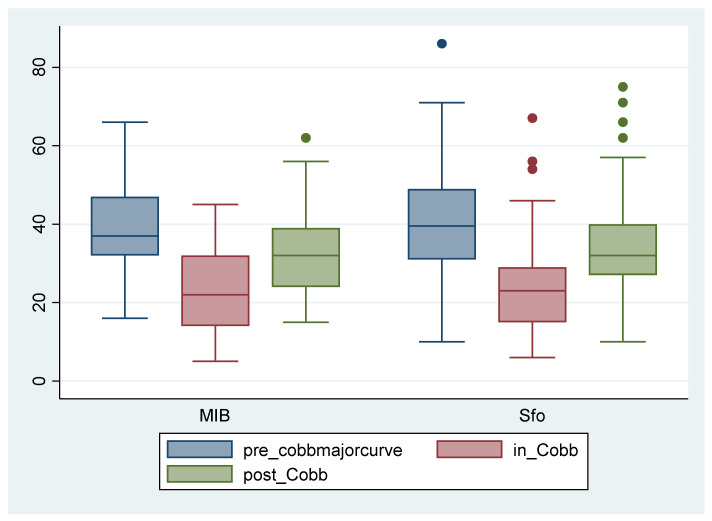
Cobb degrees of the primary curve at T0 (blue), T1 (red), and T2 (green) in the two compared groups. Sfo: Sforzesco brace; MIB: Modular Italian brace (MI brace).

**Figure 6:**
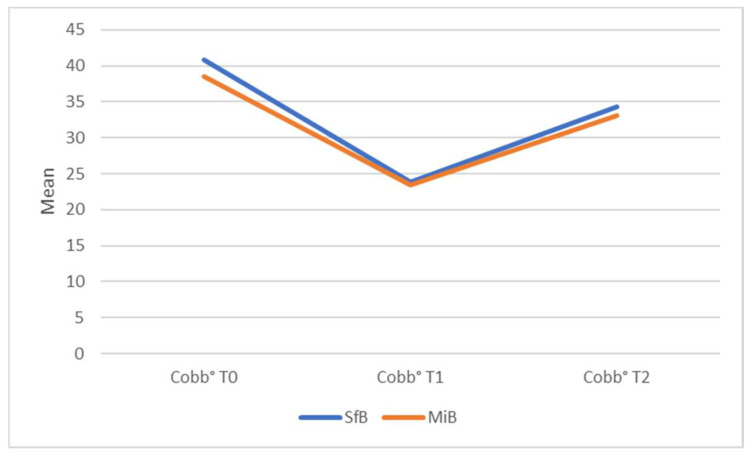
Mean Cobb degree values of primary curves at T0, T1 and T2. SfB: Sforzesco brace; MIB: Modular Italian brace (MI brace).

In-brace correction was similar with 16.9 ± 7.5° in the SfB and 16.2 ± 6.5° in the MIB group (*p* = 0.82). At T2 (first out-of-brace X-ray), both groups had a mean improvement of about 6° in Cobb angles with no significant difference between the two groups (−6.6 ± 7.6° in the SfB and −6.0 ± 7.8° in the MIB; *p* = 0.58) (Figure 5 and Figure 6). There was no significant change between groups in thoracic kyphosis (TK), lumbar lordosis (LL), TRACE, and prominence, both in ATR degrees and mm at follow-up (Table 1).

The only difference found according to brace at T2 was the proportion of improved patients; in the MIB group, more patients improved than in the SfB group. We tested the likelihood of improvement in MIB compared to SfB: patients treated with MIB were 4.9 times more likely to improve than those treated with SfB (OR = 4.92, 95%CI 1.91–12.64; *p* = 0.001). We, therefore, tested other variables: gender, age at baseline, growth, Cobb, TRACE, ATR and prominence at baseline, and sagittal parameters. The only factor that influenced the odds of improvement was the Cobb angle at baseline. After adjustment for Cobb degrees at baseline, patients in the MIB group were 5.3 times more likely to improve, and the likelihood was increased by 0.90 for each Cobb degree less at baseline (OR = 5.32; 95%CI 1.93–14.84; *p* = 0.001). 

**Figure 7:**
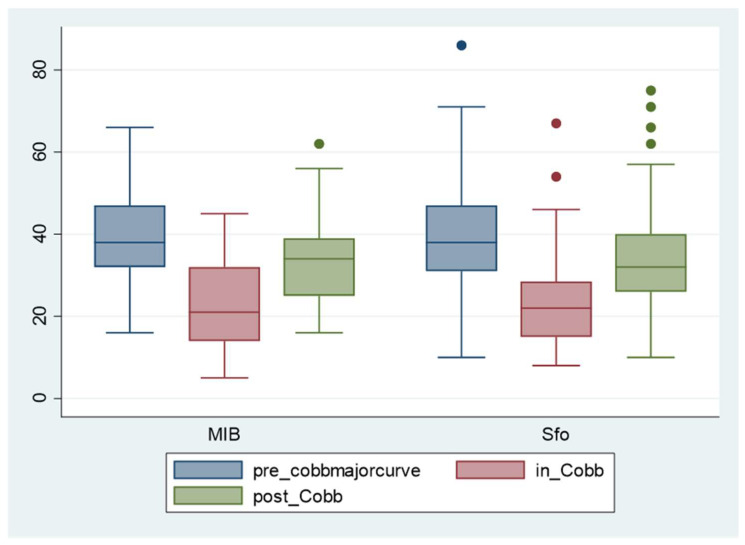
Cobb degrees of the primary curve at T0 (blue), T1 (red), and T2 (green) in the two compared groups of the subgroup including only patients starting treatment with Risser 0–2. SfB: Sforzesco brace; MIB: Modular Italian brace (MI brace).

The sensitivity analysis including only subjects with Risser 0–2 showed that there is no difference in all tested outcomes according to the brace design (Figure 7).

## 4. Discussion

The results of the present study suggest that the SfB and MIB can have similar positive short-term results on moderate-to-moderate, moderate-to-severe, and severe curves. A recent study has suggested that during bracing, the first out-of-brace radiograph could be a very strong predictor of end results [23]. For this reason, an improvement in the first out-of-brace X-ray can be considered clinically important. The MIB was prescribed with similar indications to the SfB in patients with severe scoliotic curves with the risk of becoming surgical candidates. The two compared historical groups were shown to be similar at the baseline, with the only difference being in the rate of immature patients, which was higher in the MIB group than in the SfB group. This result would suggest a more difficult population in the MIB group, even if the Cobb degrees were 2° lower than in the SfB (not significantly, either statistically or clinically). Nevertheless, we found that patients in the MIB group were 5.3 times more likely to improve and the odds of improvement were increased by 0.90 for each Cobb degree less at baseline. This suggests that the MIB may be more effective in patients with less severe scoliosis. This is an unexpected finding because the design brace is similar, with only construction differences. Hence, with this study, we were looking for similarity and not superiority of one brace. On the other hand, sensitivity analysis on younger skeletally immature patients (Risser 0–2) showed no differences between the MIB and SfB group, suggesting that the highest in odds of improvement in MIB were due to the higher number of skeletally immature patients. Further studies with different designs should clarify if this result is due to the brace or other factors. The biggest difference between the MIB and the SfB relies on its greater standardization, which could reduce the probability of mistakes from complete personalization.

Compliance was high and very similar between the two groups, with a mean of 21.7 ± 2.5 h of wearing for SfB and 21.2 ± 3.9 for MIB, and it cannot be considered a confounding factor in the present study. The high compliance for MIB is especially important; brace wearing in the first six months is strongly linked to compliance during the whole treatment [31], so the result suggests that MIB can be a well-tolerated brace.

The sagittal plane balance in spinal deformities is of the utmost importance, as misalignment can be a source of pain and disability in adult life [32,33,34]. The preservation of a balanced sagittal plane is considered one of the most important aims in AIS treatment, either conservative [11] or surgical [35,36]. According to in-brace X-rays, both brace types determine a reduction in the curvatures in the sagittal plane. Nevertheless, this reduction is temporary, and the curvature is restored after brace removal (T2). The variation is within the measurement error and is not meaningful from the clinical point of view. Furthermore, the variation was not statistically significant between the two braces. This can be related to the push-up effect, which is the correction mechanism of both braces. Studies with longer-term follow-up in larger groups will clarify if this was generated by chance or by a real difference in the sagittal action of the two braces.

The study presents some aspects of novelty and interest. Firstly, it shows the preliminary results of a different construction of a classical brace that can have many practical advantages. MIB modularity allows greater standardization, reducing the risk of mistakes and possibly allowing brace diffusion where competencies are less available for different reasons. The increased modifiability and adaptability of the brace can, on one side, allow improvement of the quality of intervention and, on the other, prolong the brace life span. All these aspects open the possibility of reducing costs and diffusing effective brace treatment in AIS patients [37]. Moreover, bracing is currently in the hands of expert orthotists, as required by SOSORT and bracing management guidelines [1]. While excellence will remain limited and required by most important and difficult cases, the modularity could facilitate the wider implementation of good bracing, reaching patients in need but currently excluded because of the unavailability of high expertise where they live. This possibility is especially important in countries of the global south, where cutting expenses is one of the most important priorities, and it can also increase compliance with treatment in patients with reduced economic means in the global north. Furthermore, the MIB personalization is less complex than building a traditional personalized brace like the SfB and requires less expertise from the technician. This can allow a faster and wider diffusion of effective braces for AIS. Finally, in the study, we present the real-life results of the treatment in an ecological context. We included more than 200 patients, reducing the possibility that the observed results are linked to random confounders. We also looked at both groups with the same treating team, reducing the source of variability due to individual choices in prescription, construction, and brace check.

Despite the study’s strengths, its conclusion should still be taken with caution. The study is retrospective, and patients treated with the SfB or MIB were not randomized. While the cohort is the same, the two groups have been treated differently in different time periods, which could lead to other factors influencing the results. The slow change from one brace to the other caused a superposition in the time of the prescriptions, and this could drive bias due to (not necessarily conscious) choices by the physician and orthotist. One possibility is that the MIB was initially prescribed more frequently in milder curves, thus introducing a selection bias, even if the baseline comparison seems to deny this possibility. Clinicians could feel less confident with the innovation and tend to protect patients with estimated higher risks by prescribing the brace they have used for longer. Nevertheless, at the baseline, the MIB was prescribed to more immature (and, consequently, at higher risk) patients. Furthermore, it should be noted that when analysing the likelihood of improvement using the MIB or SfB, the 95%CI is wide, highlighting that the variability in the sample was high, and the missing data (Table 1) could have affected these results. These findings are preliminary and should be tested in the future in larger cohorts with different study designs and longer follow-ups. Moreover, the fact that the same team treated all the patients while reducing the confounders could also lead to less generalizable results. Another potential weakness is the lack of precise data about compliance to SEAS. It is important to describe, in a detailed way, the treatment exposure of the included patients. On another hand, we decided not to consider the PSSE effect in the analysis, because PSSE has a small effect size, particularly when associated with brace treatment, as shown by Schreiber et al. comparing brace alone with brace associated with Schroth PSSE [38]. Furthermore, PSSE does not have a particularly significant effect in the short term and it is not recommended to report its effect in the short term [39]. Finally, the sample size is relatively small, particularly in the MIB group. Nevertheless, the time required to reach this sample size shows that it is not easy to gather a significant number of patients in this field, and these results should be regarded with attention due to feasibility issues.

## 5. Conclusions

In patients with severe AIS, the MIB seems to have similar, if not superior, short-term results to the SfB. These results can open up the possibility of effective brace treatment with modular braces that could lead to easier diffusion worldwide, even where there are reduced economical means and expertise on brace treatment. Before suggesting a generalized use of the MIB as a first-line rigid brace treatment, the results should be confirmed in studies with longer follow-ups. Nevertheless, it is now possible to safely substitute the SfB with the MIB without fearing reduced efficacy for patients.

## Figures and Tables

**Table 1:** Clinical and radiographical data of participants, and comparison between the two groups using unpaired T-test for continuous variables and chi-square for categorical variables. SfB: Sforzesco brace; MIB: Modular Italian brace (MI brace); SD: standard deviation; NS: not significant.

Group Comparisons	SfB Group (Total 178)		MIB (Total 53)		*p* Value (*t*-Test)
	Mean (SD)	Number of Patients	Mean (SD)	Number of Patients	
Age at start (years)	13.6 (1.7)	178	13.5 (1.9)	53	NS
Age at the end (years)	14.1 (1.7)	178	14.0 (2.0)	47	NS
Baseline Cobb angle (T0) (Cobb °)	40.9 (12.8)	178	38.6 (11.2)	53	NS
In-brace Cobb angle (T1) (Cobb °)	23.8 (10.8)	122	23.4 (10.7)	41	NS
First out-of-brace X-ray Cobb angle (T2) (Cobb °)	34.3 (11.5)	177	33.1 (11.2)	47	NS
In brace correction (Cobb °)	16.9 (7.5)	122	16.2 (6.5)	41	NS
Cobb change from baseline to short term follow up (Cobb °)	6.6 (7.5)	177	6.0 (7.8)	53	NS
Cobb angle loss from in-brace to out-of-brace (Cobb °)	9.6 (5.8)	122	9.3 (5.8)	38	NS
Thoracic kyphosis change (T1–T12) (Cobb °)	−6.6 (0.6)	177	−6.0 (7.8)	47	NS
Lumbar lordosis change (T12–L5) (Cobb °)	−3.1 (10.6)	69	−0.6 (8.6)	32	NS
Daily brace wear (hours)	21.7 (2.5)	178	21.2 (3.9)	44	NS
Baseline ATR (degrees)	11.4 (4.5)	177	10.9 (5.0)	53	NS
Baseline prominence (mm)	18.7 (8.7)	177	17.0 (8.5)	53	NS
Baseline TRACE	7.8 (2.2)	178	7.5 (1.9)	53	NS
ATR change (°)	−3.0 (3.7)	175	−2.9 (5.1)	48	NS
Prominence change (mm)	−4.7 (6.5)	175	−4.4 (7.7)	47	NS
TRACE change	−3.1 (2.3)	178	−3.0 (2.2)	47	NS
Growth (cm)	3.2 (8.3)	172	3.2 (2.0)	46	NS
Time of follow up (years)	1.2 (0.7)	178	1.4 (1.2)	47	NS
	Number	Proportion	Number	Proportion	Chi-square (P)
Gender: male (n)	26	14.6%	13	24.5%	2.8649 (NS)
female	152	85.4%	40	75.5%	
Risser: 0 (n)	51	28.8%	17	32.1%	6.071 (NS)
1	34	19.2%	17	32.1%	
2	31	17.5%	8	15.1%	
3	47	26.6%	9	17.0%	
4–5	14	7.9%	2	3.8%	
Improved (≥5 Cobb°) (n)	9	5.1%	11	20.7%	12.73 (*p* < 0.001)
	Improved patients were significantly higher in MIB				
Worsened ≥ 5 Cobb° (n)	75	42.1%	23	43.4%	0.0266 (NS)
Below 30 Cobb (n)	73	41.0%	22	41.5%	0.0042 (NS)
Above 45 Cobb (n)	29	16.3%	13	24.5%	1.86 (NS)

## Data Availability

The data presented in this study are openly available from Zenodo at https://zenodo.org/records/10445142 (accessed on 30 December 2023).

## References

[1] Negrini S., Donzelli S., Aulisa A.G., Czaprowski D., Schreiber S., de Mauroy J.C., Diers H., Grivas T.B., Knott P., Kotwicki T. (2018). 2016 SOSORT Guidelines: Orthopaedic and Rehabilitation Treatment of Idiopathic Scoliosis during Growth. Scoliosis Spinal Disord.

[2] Weinstein S.L., Dolan L.A., Cheng J.C.Y., Danielsson A., Morcuende J.A. (2008). Adolescent Idiopathic Scoliosis. Lancet.

[3] Hresko M.T. (2013). Clinical Practice. Idiopathic Scoliosis in Adolescents. N. Engl. J. Med..

[4] Weinstein S.L., Dolan L.A., Wright J.G., Dobbs M.B. (2013). Effects of Bracing in Adolescents with Idiopathic Scoliosis. N. Engl. J. Med..

[5] Aulisa A.G., Toniolo R.M., Falciglia F., Giordano M., Aulisa L. (2021). Long-Term Results after Brace Treatment with Progressive Action Short Brace in Adolescent Idiopathic Scoliosis. Eur. J. Phys. Rehabil. Med..

[6] Negrini S., Donzelli S., Negrini F., Arienti C., Zaina F., Peers K. (2021). A Pragmatic Benchmarking Study of an Evidence-Based Personalised Approach in 1938 Adolescents with High-Risk Idiopathic Scoliosis. J. Clin. Med..

[7] Guo J., Lam T.P., Wong M.S., Ng B.K.W., Lee K.M., Liu K.L., Hung L.H., Lau A.H.Y., Sin S.W., Kwok W.K. (2014). A Prospective Randomized Controlled Study on the Treatment Outcome of SpineCor Brace versus Rigid Brace for Adolescent Idiopathic Scoliosis with Follow-up According to the SRS Standardized Criteria. Eur. Spine J..

[8] Charalampidis A., Diarbakerli E., Dufvenberg M., Jalalpour K., Ohlin A., Ahl A.A., Möller H., Abbott A., Gerdhem P., CONTRAIS Study Group (2024). Nighttime Bracing or Exercise in Moderate-Grade Adolescent Idiopathic Scoliosis: A Randomized Clinical Trial. JAMA Netw. Open.

[9] Guy A., Labelle H., Barchi S., Audet-Duchesne E., Cobetto N., Parent S., Raison M., Aubin C.-É. (2021). Braces Designed Using CAD/CAM Combined or Not With Finite Element Modeling Lead to Effective Treatment and Quality of Life After 2 Years: A Randomized Controlled Trial. Spine.

[10] Negrini S., Minozzi S., Bettany-Saltikov J., Chockalingam N., Grivas T.B., Kotwicki T., Maruyama T., Romano M., Zaina F. (2015). Braces for Idiopathic Scoliosis in Adolescents. Cochrane Database Syst. Rev..

[11] Negrini S., Aulisa A.G., Cerny P., de Mauroy J.C., McAviney J., Mills A., Donzelli S., Grivas T.B., Hresko M.T., Kotwicki T. (2022). The Classification of Scoliosis Braces Developed by SOSORT with SRS, ISPO, and POSNA and Approved by ESPRM. Eur. Spine J..

[12] Tang S., Cheung J.P.Y., Cheung P.W.H. (2024). Effectiveness of Bracing to Achieve Curve Regression in Adolescent Idiopathic Scoliosis. Bone Jt. J..

[13] Del Prete C.M., Tarantino D., Viva M.G., Murgia M., Vergati D., Barassi G., Sparvieri E., Di Stanislao E., Perpetuini D., Russo E.F. (2023). Spinal Orthosis in Adolescent Idiopathic Scoliosis: An Overview of the Braces Provided by the National Health Service in Italy. Medicina.

[14] Liu S., Ho L.Y., Hassan Beygi B., Wong M.S. (2023). Effectiveness of Orthotic Treatment on Clinical Outcomes of the Patients with Adolescent Idiopathic Scoliosis Under Different Wearing Compliance Levels: A Systematic Review. JBJS Rev..

[15] Negrini S., Tessadri F., Negrini F., Tavernaro M., Zonta A., Zaina F., Donzelli S. (2022). Impact of the Free-Pelvis Innovation in Very Rigid Braces for Adolescents with Idiopathic Scoliosis: Short-Term Results of a Matched Case-Control Study. Children.

[16] Lou E., Ng K., Hill D. (2022). Immediate Outcomes and Benefits of 3D Printed Braces for the Treatment of Adolescent Idiopathic Scoliosis. Front. Rehabil. Sci..

[17] Rothstock S., Weiss H.-R., Krueger D., Kleban V., Paul L. (2020). Innovative Decision Support for Scoliosis Brace Therapy Based on Statistical Modelling of Markerless 3D Trunk Surface Data. Comput. Methods Biomech. Biomed. Eng..

[18] Wong M.S., Beygi B.H., Wong K.W., Sin S.W., Kwok W.K., Wu H.D. (2022). Effect of Different Undergarment Designs on the Compliance and Acceptance of the Patients with Adolescent Idiopathic Scoliosis under Orthotic Treatment. Prosthet. Orthot. Int..

[19] Lou E., Hill D., Raso J., Donauer A., Moreau M., Mahood J., Hedden D. (2012). Smart Brace versus Standard Rigid Brace for the Treatment of Scoliosis: A Pilot Study. Stud. Health Technol. Inform..

[20] Nagy Szabó O., Geršak J., Koleszár A., Halász M. (2023). Influence of Undergarments on the Comfort Level of Scoliosis Brace Wearers. Materials.

[21] Rahimi S., Kiaghadi A., Fallahian N. (2020). Effective Factors on Brace Compliance in Idiopathic Scoliosis: A Literature Review. Disabil. Rehabil. Assist. Technol..

[22] Nathan P., Chou S.M., Liu G. (2023). A Review on Different Methods of Scoliosis Brace Fabrication. Prosthet. Orthot. Int..

[23] Negrini S., Di Felice F., Negrini F., Rebagliati G., Zaina F., Donzelli S. (2022). Predicting Final Results of Brace Treatment of Adolescents with Idiopathic Scoliosis: First out-of-Brace Radiograph Is Better than in-Brace Radiograph-SOSORT 2020 Award Winner. Eur. Spine J..

[24] von Elm E., Altman D.G., Egger M., Pocock S.J., Gøtzsche P.C., Vandenbroucke J.P., STROBE Initiative (2007). The Strengthening the Reporting of Observational Studies in Epidemiology (STROBE) Statement: Guidelines for Reporting Observational Studies. Lancet.

[25] Berdishevsky H., Lebel V.A., Bettany-Saltikov J., Rigo M., Lebel A., Hennes A., Romano M., Białek M., M’hango A., Betts T. (2016). Physiotherapy Scoliosis-Specific Exercises—A Comprehensive Review of Seven Major Schools. Scoliosis Spinal Disord..

[26] Karavidas N., Tzatzaliaris D. (2022). Brace and Physiotherapeutic Scoliosis Specific Exercises (PSSE) for Adolescent Idiopathic Scoliosis (AIS) Treatment: A Prospective Study Following Scoliosis Research Society (SRS) Criteria. Arch. Physiother..

[27] Negrini S., Marchini G., Tessadri F. (2011). Brace Technology Thematic Series—The Sforzesco and Sibilla Braces, and the SPoRT (Symmetric, Patient Oriented, Rigid, Three-Dimensional, Active) Concept. Scoliosis.

[28] Dolan L.A., Donzelli S., Zaina F., Weinstein S.L., Negrini S. (2020). Adolescent Idiopathic Scoliosis Bracing Success Is Influenced by Time in Brace: Comparative Effectiveness Analysis of BrAIST and ISICO Cohorts. Spine.

[29] Negrini S., Donzelli S., Di Felice F., Zaina F., Caronni A. (2020). Construct Validity of the Trunk Aesthetic Clinical Evaluation (TRACE) in Young People with Idiopathic Scoliosis. Ann. Phys. Rehabil. Med..

[30] Donzelli S., Zaina F., Negrini S. (2012). In Defense of Adolescents: They Really Do Use Braces for the Hours Prescribed, If Good Help Is Provided. Results from a Prospective Everyday Clinic Cohort Using Thermobrace. Scoliosis.

[31] Linden G.S., Emans J.B., Karlin L.I., O’Neill N.P., Williams K.A., Hresko M.T. (2023). Early Adherence to Prescribed Brace Wear for Adolescent Idiopathic Scoliosis Is Associated With Future Brace Wear. Spine.

[32] Maria C.-W., Patryk W., Mateusz Ż., Marcin T. (2023). Is Sagittal Spinopelvic Alignment a Cause of Low Back Pain in Pediatric Spine Pathologies? A Review. J. Child. Orthop..

[33] Kyrölä K., Repo J., Mecklin J.-P., Ylinen J., Kautiainen H., Häkkinen A. (2018). Spinopelvic Changes Based on the Simplified SRS-Schwab Adult Spinal Deformity Classification: Relationships With Disability and Health-Related Quality of Life in Adult Patients With Prolonged Degenerative Spinal Disorders. Spine.

[34] Smith J.S., Klineberg E., Schwab F., Shaffrey C.I., Moal B., Ames C.P., Hostin R., Fu K.-M.G., Burton D., Akbarnia B. (2013). Change in Classification Grade by the SRS-Schwab Adult Spinal Deformity Classification Predicts Impact on Health-Related Quality of Life Measures: Prospective Analysis of Operative and Nonoperative Treatment. Spine.

[35] Rubery P.T., Lander S.T., Mesfin A., Sanders J.O., Thirukumaran C.P. (2022). Mismatch Between Pelvic Incidence and Lumbar Lordosis Is the Key Sagittal Plane Determinant of Patient Outcome at Minimum 40 Years After Instrumented Fusion for Adolescent Idiopathic Scoliosis. Spine.

[36] Thomas E.S., Boyer N., Meyers A., Aziz H., Aminian A. (2023). Restoration of Thoracic Kyphosis in Adolescent Idiopathic Scoliosis with Patient-Specific Rods: Did the Preoperative Plan Match Postoperative Sagittal Alignment?. Eur. Spine J..

[37] Ikwuezunma I., Wang K., Margalit A., Sponseller P., Jain A. (2021). Cost-Utility Analysis Comparing Bracing Versus Observation for Skeletally Immature Patients with Thoracic Scoliosis. Spine.

[38] Schreiber S., Parent E.C., Khodayari Moez E., Hedden D.M., Hill D.L., Moreau M., Lou E., Watkins E.M., Southon S.C. (2016). Schroth Physiotherapeutic Scoliosis-Specific Exercises Added to the Standard of Care Lead to Better Cobb Angle Outcomes in Adolescents with Idiopathic Scoliosis—An Assessor and Statistician Blinded Randomized Controlled Trial. PLoS ONE.

[39] Negrini S., Hresko T.M., O’Brien J.P., Price N. (2015). Recommendations for Research Studies on Treatment of Idiopathic Scoliosis: Consensus 2014 between SOSORT and SRS Non–Operative Management Committee. Scoliosis.

